# Performance Assessment of Reference Modelling Methods for Defect Evaluation in Asphalt Concrete

**DOI:** 10.3390/s21248190

**Published:** 2021-12-08

**Authors:** Pauli Putkiranta, Matti Kurkela, Matias Ingman, Aino Keitaanniemi, Aimad El Issaoui, Harri Kaartinen, Eija Honkavaara, Hannu Hyyppä, Juha Hyyppä, Matti T. Vaaja

**Affiliations:** 1 Department of Built Environment, Aalto University, 02150 Espoo, Finland; matti.kurkela@aalto.fi (M.K.); matias.ingman@aalto.fi (M.I.); aino.keitaanniemi@aalto.fi (A.K.); hannu.hyyppa@aalto.fi (H.H.); matti.t.vaaja@aalto.fi (M.T.V.); 2 Department of Remote Sensing and Photogrammetry, Finnish Geospatial Research Institute FGI, The National Land Survey of Finland, Geodeetinrinne 2, 02430 Masala, Finland; aimad.elissaoui@nls.fi (A.E.I.); harri.kaartinen@nls.fi (H.K.); eija.honkavaara@nls.fi (E.H.); juha.hyyppa@nls.fi (J.H.); 3 Department of Geography and Geology, University of Turku, 20500 Turku, Finland

**Keywords:** laser scanning, photogrammetry, road maintenance, pavement defects, structured light, reference measurements

## Abstract

The deterioration of road conditions and increasing repair deficits pose challenges for the maintenance of reliable road infrastructure, and thus threaten, for example, safety and the fluent flow of traffic. Improved and more efficient procedures for maintenance are required, and these require improved knowledge of road conditions, i.e., improved data. Three-dimensional mapping presents possibilities for large-scale collection of data on road surfaces and automatic evaluation of maintenance needs. However, the development and, specifically, evaluation of large-scale mobile methods requires reliable references. To evaluate possibilities for close-range, static, high-resolution, three-dimensional measurement of road surfaces for reference use, three measurement methods and five instrumentations are investigated: terrestrial laser scanning (TLS, Leica RTC360), photogrammetry using high-resolution professional-grade cameras (Nikon D800 and D810E), photogrammetry using an industrial camera (FLIR Grasshopper GS3-U3-120S6C-C), and structured-light handheld scanners Artec Leo and Faro Freestyle. High-resolution photogrammetry is established as reference based on laboratory measurements and point density. The instrumentations are compared against one another using cross-sections, point–point distances, and ability to obtain key metrics of defects, and a qualitative assessment of the processing procedures for each is carried out. It is found that photogrammetric models provide the highest resolutions (10–50 million points per m^2^) and photogrammetric and TLS approaches perform robustly in precision with consistent sub-millimeter offsets relative to one another, while handheld scanners perform relatively inconsistently. A discussion on the practical implications of using each of the examined instrumentations is presented.

## 1. Introduction

Road networks require large investments for construction, renewal and maintenance. Growing networks demand increasing investments while repair deficits continue to grow. In 2017, the repair deficit of the Finnish road network was approximately EUR 1.3 billion [1], while in the USA, it was USD 420 billion [2]. In the European Union, deficits have grown since investments dwindled after the 2008 financial crisis, though exact figures are unavailable [3]. Materially, deficits translate into pavement defects, caused by weathering, wear, and structural problems, which in turn decrease safety, disturb traffic flow, increase fuel use, and cause time delays and discomfort [4].

Hadjidemetriou et al. [5] outline four “diseases”, or distress types, for pavement defect classification, presented in Table 1. In Finnish conditions, the Finnish Transport Infrastructure Agency [6] explains most of these defects, especially rutting and cracking, with climatic reasons, specifically the use of studded tires in the winter and water freezing under the pavement. In Nordic conditions, rutting has been explored by Lampinen [7], while Belt et al. [8] have modeled the structural deterioration and predicted future condition of roads. Automation and high-precision instrumentation can provide high-quality data on road pavement conditions, allowing for accurate estimation of the maintenance needs and prioritization of different targets.

Comprehensive knowledge about road conditions is necessary for planning timely maintenance procedures [9,10]. However, many road quality surveying methods that are currently in use appear out of date. Rutting is often measured using laser profilometers with limited numbers of lasers, e.g., 17 [11,12,13], while cracking and potholes are identified manually or using low-resolution images and rudimentary feature extraction algorithms on limited areas [14]. Better coverage and more information on road quality allow for more effective pavement management [15]. In fact, many proposals for crack and distress detection have been made in recent years, as can be seen in multiple reviews [16,17,18,19]. In addition to detection and identification, some degree of information extraction is necessary to determine the need for maintenance procedures. While some information can be extracted from 2D images, 3D—and with time series, even 4D—data provide opportunities for more accurate assessments.

Distress detection and analysis is only one aspect of effective pavement management. In addition to pavement surface conditions, effective management requires knowledge about the foundations of the road, such as structure bearing capacity and pavement thickness [20]. While surface deformations and distress can reveal structural issues [21], dedicated tools such as ground-penetrating radar and deflectometers are important in establishing underlying structural conditions [22,23]. In some cases, embedded sensors are used to continuously monitor structural conditions [24]. Through the use these kinds of methods, possible structural failures can be predicted [22]. While possibilities for structural evaluation beyond the visible surface exist, surface distress detection is an essential part of a viable pavement management system [15]. Indeed, an effective approach integrates different data sources to continuously monitor a road through its lifetime, model structural responses to use, evaluate performance, and prompt maintenance [24].

The development of practically feasible and efficient—mobile, automated—distress detection methods requires accurate information about road surfaces and defects. Defects vary in size, ranging from small cracks to large potholes. At the same time, asphalt concrete surfaces are relatively coarse, and distinguishing small cracks can be challenging, depending on resolution and precision. High-resolution reference models allow the identification and quantification of both small and large defects. These reference models can then be used in the development of automated methods for defect detection, measurement, and classification. However, the production of high-resolution models can be laborious and time-consuming. This article aims to compare and contrast various methods for the production of such reference models in realistic circumstances, where conditions are nonoptimal.

Point clouds are simple and common 3D model formats, and can be produced by measurements with, for example, laser scanners and photogrammetry. Evaluating the quality of point clouds produced by different methods is fundamentally a comparative endeavor. Many different approaches have been proposed for comparing point clouds. Lehtola et al. [25] divide point cloud quality evaluation into three approaches: (1) the control point approach, where a distance between two control points is evaluated from different point clouds; (2) the subset approach, where a subset of a point cloud is extracted and evaluated, for example, by comparing the planarity of a subset representing a wall; and (3) the full point cloud approach, where point clouds are taken in their entirety and compared using an arbitrary metric. The nature of pavement defects and their measurement places the evaluation of their quality in the second—subset—approach, since it makes sense to extract, compare, and evaluate individual defects or areas of interest. Different aspects of, and approaches to, point cloud quality have been investigated by many authors, for example quality metrics [26,27], subjective assessment [28,29], interactive evaluation [30], and color [31,32].

Considering the geometric accuracy of a given point cloud poses a challenge, as this is typically done by comparing the cloud to another, one of greater accuracy, and deviation determines the accuracy of the given cloud. Without an established reference modeling method, there is no obvious way to determine ground truth. Nonetheless, comparisons allow us to determine deviations between modeling methods and to establish any systematic failures in instruments. Additionally, the use of established and well-calibrated methods, such as terrestrial laser scanning (TLS), which can be considered accurate within its calibrated accuracy, provides a reliable baseline [33]. However, TLS data may be too sparse for accurate quantification of pavement defects. Various approaches have previously been used for pavement modeling. Inzerillo et al. [34] modeled a large pothole using both TLS and handheld photogrammetry, determining that the photogrammetric model can be of higher precision. Knyaz and Chibunichev [35] constructed a stereo camera system that uses structured light and used it to model the deformation of a paved surface, reporting the measuring accuracy to be 0.1 mm, and 3D model resolution about 0.3 mm. Puzzo et al. [36] determined the accuracies of various cameras in photogrammetric modeling of asphalt surfaces for roughness modeling, concluding that digital single-lens reflex (dSLR) cameras outperform others.

This research examines three technologies (photogrammetry, terrestrial laser scanning, and structured-light laser scanning with handheld scanners) and five instrumentations for creating high-density and highly accurate point clouds of road surfaces for referential use in the development of automated defect detection and analysis systems. These instrumentations, described in this article as methods, are examined in realistic circumstances; that is, test plots are real road surfaces, defects are real defects, and measurements are conducted alongside traffic in natural lighting and nonoptimal weather conditions. Compromises were made in measurements, and obtained measurements are nonideal. As a result, results are nonuniversal, but provide a comparative case study of how different approaches to road surface modeling perform. For ground truth, laboratory measurements with pavement samples are conducted. A state-of-the-art TLS instrument is contrasted with two photogrammetric approaches and two handheld structured-light scanners. Based on reference measurements, photogrammetry based on high-resolution images is chosen as a reference for further evaluation of other methods. Point clouds are compared directly by utilizing cross-sections, other visualizations, and point–point distances between clouds, and indirectly by comparing volume and maximum depth of defects as measured by the different instruments.

## 2. Materials and Methods

The measurements for this research were conducted in three separate settings. The main contribution of this study consists of field measurements with various instrumentations and analysis on quantitative and qualitative difference between different approaches from the perspective of their use as reference measurements. In addition to field measurements, ground truth is established through the use of pavement samples that are measured in laboratory conditions. In order to transfer this reference setup to realistic conditions, control measurements are conducted using the same samples outdoors, in conditions corresponding to field measurements on active roads. This section describes the instruments that are being compared, as well as presenting the various measurement scenarios. First, we present the test site and research plots where measurements were conducted; second, we present instruments and how data is processed in each case; third, we present the reference measurement setup in the laboratory and how this is generalized to field measurements; finally, we present the quantitative and qualitative analysis that is conducted to assess each instrument.

### 2.1. Test Site

**Figure 1:**
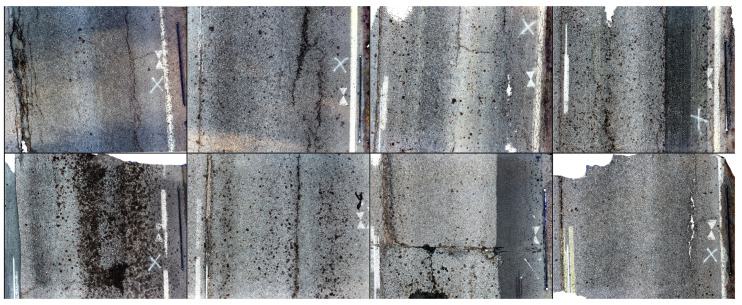
Mosaic composed of orthoimages of examined 8 plots, top row plots 1–4, bottom row plots 5–8. The black stick with white targets that is visible in most images measures 2 m.

Measurements were conducted in the summer of 2020, on eight selected plots of Masalantie in Kirkkonummi, near Helsinki, Finland. The plots, pictured in Figure 1 and identified in Table 2, were selected for quantity of, and variety in, pavement defects. They are approximately 4 (width) × 3 (length) meters in size. Single defects (cracks, potholes, deteriorations) were sectioned out of plots to increase the number of research areas and because the modeling of these defects is of primary interest in this research, rather than the modeling of nondamaged pavement.

### 2.2. Instruments and Data Processing

Three methods and five instrumentations were employed in field measurements: high-resolution photogrammetry, industrial camera photogrammetry, terrestrial laser scanning, and two handheld structured-light scanners (Artec Leo and Faro Freestyle).

#### 2.2.1. High-Resolution Photogrammetry

Two cameras were used: Nikon D800E with a Nikkor AF-S 14-24 mm f/2.8 G lens locked at 24 mm, and Nikon D810 with a Nikkor AF-S 50 mm f/1.4 G and Nikkor AF-S 60 mm f/2.8 G Micro objectives. The 60 mm lens was used on plots 1–2 and the 50 mm lens on plots 3–8. Reference measurements were made using the 50 mm lens. Both cameras use an FX format 35.9 × 24.0 mm CMOS sensor. Image size in pixels is 7360 × 4912. Table 3 describes the settings used in cameras.

For field imaging, the camera was handheld at approximately 1.7 m from the road surface, i.e., face level. The imaging geometry was designed to be slightly convergent—that is, the camera was slightly tilted at different angles from the vertical in a locally near-parallel manner—in order to mitigate systematic errors and prevent deformation as explained by James and Robson [37]. Imaging was carried out under mostly diffuse illumination conditions in the late evening and early morning to avoid traffic and direct sunlight. Thus, lighting conditions also changed accordingly as the sun set and rose, and this is apparent in ISO and shutter speed settings (Table 3). Some 170–350 images were taken of each plot (see Table 2), with specific attention paid to photographing any defects as thoroughly as possible. Ground sampling distance with the 50 mm lens was approximately 0.16–0.17 mm/px.

The images were aligned and processed into point clouds using RealityCapture [38]. RealityCapture allows the images to be processed alongside laser scanning data, which provides georeferencing information. Attention was paid to achieve the highest quality possible. RealityCapture processing settings are shown in Table 4. High-resolution photogrammetry is also referred to as Nikon photogrammetry in this paper, for brevity.

#### 2.2.2. Industrial Camera Photogrammetry

Images were also collected using a Grasshopper3 USB3 industrial camera (model: GS3-U3-120S6C-C, Teledyne FLIR LLC, Wilsonville, OR, USA). All plots were photographed using a 1:1.4/12.5 mm Fujinon CF12.5HA-1 lens. Imaging was carried out manually by holding the camera at approximately a height of 1 m while walking through the plot in a back-and-forth manner. The camera was set to collect raw sensor data at 7 FPS, with aperture value 8, gain 2.0, and automatic shutter speed. Approximately 500–1500 images resulted from each plot (see Table 2. The large variance reflects the fact that measurements were made manually at walking speed, and gait varied, and occasionally it was necessary to wait for vehicles to pass for safety reasons. Ground sampling distance was approximately 0.25 mm.

Grasshopper (GH) imaging did not pay special attention to defects in the pavement surface. Rather, the imaging method was designed to correspond to a rig of multiple adjacent cameras sweeping the pavement surface, for example, installed onto a car. Of course, walking speed is much slower than typical driving speed, meaning that images collected from a car might have more motion blur and the amount of collected images was large. The images were then aligned and processed into dense point clouds in Agisoft Metashape [39] software, using parameters presented in Table 5.

#### 2.2.3. Terrestrial Laser Scanning

TLS data collection was conducted using a Leica RTC360 scanner [40]. Two scans were taken from each plot, one on each side of the plot, moving in the direction of the road. For each scan, the medium setting was used, corresponding to point resolution of 6 mm at a distance of 10 m [41]. As the distance between the two scan locations was significantly smaller, the point resolution was expected to be higher in the plot area. Images were taken with the integrated camera of the scanner for coloring the point cloud (these images were also used in georeferencing photogrammetric datasets), and each point was scanned twice in order to ensure a correct return.

Using the aforementioned settings, each scan took roughly four minutes, when the scanner captured a 360° horizontal area and a 300° vertical area. It was later noted that the scan time could be reduced by limiting the scan to the plot area, as the plots were the only area of interest. In order to georeference the point cloud, three target spheres on tripods were used at each plot, and GNSS measurements were taken from each.

The scan data for each plot were registered in Leica Register 360 [42], where the point clouds were automatically registered to each other using cloud-to-cloud alignment and finding target spheres in both scans. The automatic registration can be improved through manual alignment and reregistration in Register 360, if necessary, though with two scans and the use of target spheres, the registration was found to be sufficiently accurate as provided. Thus, the registration process was conducted automatically, with the eight plots requiring a total of two hours to register. The coordinates for the target spheres were also entered in Register 360 to georeference the point clouds. The point clouds were then segmented manually in CloudCompare 2.10.2 to separate the plots from the other areas of the scan, as only the plot areas are considered in this study. The radiometric qualities of the RTC360 have been examined in [31].

#### 2.2.4. Artec Leo

Test plots were modeled using the Artec Leo handheld scanner [43] (see specifications in Table 6). Due to practical reasons, measurements with the Leo were made 2.5 months later than other measurements, in August 2020. The plots were scanned by holding the scanner at waist height (approximately 0.9 meters), pointing it at the pavement, and walking through the area in a back-and-forth manner. Measurements were made on an overcast day, as sunlight disrupts the structured-light pattern of the scanner. The time difference between Leo and other measurements means that some deterioration in the pavement may have occurred in the meantime.

Scans were processed using Artec’s proprietary Artec Studio 15 software [44]. The software has some black box functionalities, but processing mainly consists of the following steps: alignment/registration; outlier removal; model “fusion”; and texturing. The qualitative experience is that resulting models are rather smooth, and planar surfaces—lacking in 3D features—are challenging for the system. As will be seen later, the Leo occasionally lost track of the surface and created slightly deformed (curved) surfaces in general.

#### 2.2.5. Faro Freestyle

The test plots were scanned with the Faro Freestyle (FF) [45] handheld scanner, which includes two infrared cameras, a color camera, and a laser unit. Some specifications are available in Table 6. The measurement principle of the scanner is based on structured-light technology, in which two infrared cameras are stereo-imaging a structured-light pattern formed on a surface. Since sunlight interferes with the infrared sensors’ ability to detect the pattern, measurement was performed at dawn, when there was enough light for the color camera and minimal traffic flow. The measurement was performed by placing calibration signs around the defects in each plot, after which the test area was scanned only from the area around the calibration signs. The measurement was performed at a distance of one meter from the ground surface by walking and by closing the loop, meaning that the scanning process was begun and finished at the same point. In this way, it was ensured that the scans were matched as well as possible and that the defects could be scanned from at least two directions.

The point clouds were processed in the Faro Scene Process [46], where automatic processing was performed for data optimization. During processing, the Faro Scene Process combines Freestyle’s multiple scanframes into a single point cloud.

### 2.3. Reference Measurements

#### 2.3.1. Pavement Samples

**Figure 2:**
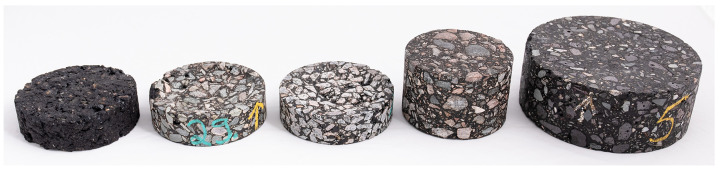
Pavement samples used for reference measurement. Samples in ID number order, from left to right, 1–5.

In order to define a standard for comparing the instruments in the test site, as described above, standard pavement samples were used to establish ground truth. A collection of five different kinds of samples was used, with varying degrees of wear and various textures. These are pictured in Figure 2 and described in Table 7.

#### 2.3.2. Reference Measurement Setup

The samples were first measured using laboratory-grade structured-light scanner Konica Minolta VIVID 9i Non-Contact 3D Digitizer. As per recommendations [47], measurements were conducted indoors at room temperature, and natural light was blocked. Calibration was conducted using the Field Calibration System for 744 mm TELE lens and 694 mm MIDDLE lens—the latter of which was need for the largest pavement sample. Scanning mode was set to standard, and number of scans to four. Scans were saved using Polygon Editing Tool [48] software with no hole filling and 1:1 reduction rate. Mesh models were saved as OBJ files.

**Figure 3:**
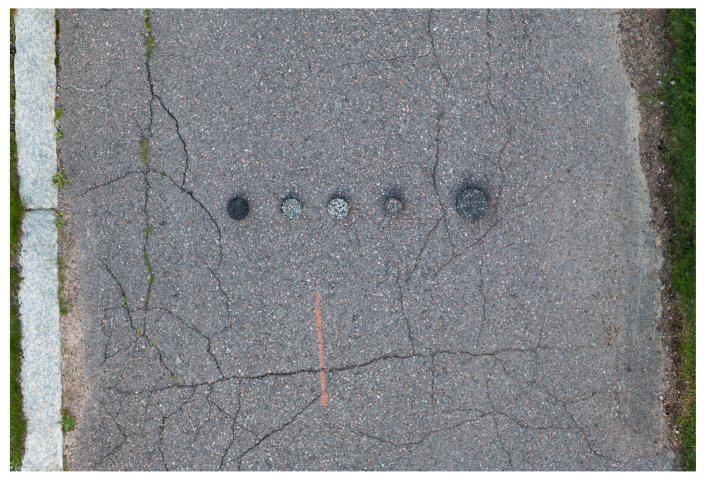
Reference measurement setup. Samples are in ID order 1–5 from left to right.

After laboratory measurements, the pavement samples were placed in a row on a paved area outdoors in order to simulate realistic measurement conditions. Then measurements were conducted with each instrument as described above. No special attention was paid to the samples. The reference measurement test site is pictured in Figure 3.

#### 2.3.3. Reference Measurement Analysis

After data processing, resulting point clouds and meshes were registered in CloudCompare [49] (using georeferenced TLS and photogrammetric measurements to georeference other datasets). Registration was done manually at first and refined using the iterative closest point (ICP) algorithm. Point clouds were manually segmented to only cover areas that are included in reference meshes—that is, only the top surfaces of all samples are included. Instruments were then compared against ground truth as established by the laboratory scanner using point–point distances and standard deviations between individual samples. Additionally, the number of points in the segmented clouds was observed to indicate point cloud density.

### 2.4. Data Analysis

The various colored point clouds were aligned first manually and then using ICP in CloudCompare. The universally (across the entire test plot) aligned clouds were exported. Individual defects were also sectioned from the point clouds manually using bounding boxes and exported.

#### 2.4.1. Plot-Level and Cross-Section Analysis

For a universal comparison, the point clouds were imported into Matlab [50] and visualizations were made of overlaid clouds and cross sections. These were used for visual comparisons of entire test plots. Cross sections can be used to identify discontinuities, noise levels, and possible drift in point clouds. Visualizations of entire plots are often difficult to interpret, especially if multiple point clouds are overlaid. More detailed comparisons were made on the defect level.

#### 2.4.2. Defect-Level Analysis: Point–Point Distances

On the single defect level, clouds were compared quantitatively using point–point distances in CloudCompare, in addition to which some visualizations were produced to gain qualitative understanding of the differences between various modeling methods. While numbers reveal general levels of precision, visualizations help explain the reasons for any particular offsets.

To quantitatively compare the different point clouds, it is necessary to establish a reference point cloud that other clouds are compared to. In this study, the photogrammetric point clouds created from Nikon images are used as references. This is based on reference results and the fact that such photogrammetric modeling is an established method for creating high-detail models [37]. Additionally, it can be visually confirmed that the photogrammetric point clouds closely match the TLS point clouds, which can be assumed to be geometrically accurate, while having higher point density and less noise (see Section 3.4).

However, since the photogrammetric method does not represent a real ground truth, other methods can also be cross-compared. In this fashion, it is possible to examine the other methods in a broader sense and understand more completely how the different methods compare to each other. It also enables us to question and evaluate the validity of the photogrammetric model as reference. This is why point–point distance comparisons are made across all methods and evaluated holistically.

#### 2.4.3. Defect Analysis: Volume and Maximum Depth

Some characteristics of defects were also calculated and used to categorize the defects and further evaluate the possibilities of different sensors. In the geometric domain, measuring various properties such as length, width, area, and volume is essential in determining the severity of a defect as well as the maintenance needs. Defects that cover a large area are more susceptible to warping, which may happen when modeling planar surfaces. On the other hand, more voluminous defects have stronger (i.e., less planar) geometry, which might make them easier to model. In addition, defect volume can be a useful metric for evaluating the need for maintenance.

The area covered by a defect can be roughly calculated by simply multiplying the length of two sides of the manually-defined bounding box by which the defect is defined. Of course, some of this area includes nondefective pavement, but since all of the area is used to evaluate the point–point distances between point clouds, it is reasonable to include it entirely. If another defect intersects with this area, the defect is discluded. In practice, there is not always a clear delineation between defects or between defect and nondefective pavement, and it is a matter of judgment to define the limits of a defect. To calculate the volume of a defect, a plane can be fitted on the surface around the defect and then the volume between the plane and the defect can be calculated. In CloudCompare, the plane is fit using the Fit Plane tool (a least-squares approach) and volume calculated using the 2.5D Volume tool. It should be noted that road surfaces are not planar due to, for example, rutting. This means that, especially in the case of defects with large surface areas, an ideal plane may not correspond to the surface. At the same time, it could be argued that the effect of this on the volume computation might be counteracted by differences in volume due to nonplanarity being systematic, which means that as much volume should be added as is removed by the effect of nonplanarity.

Volume, as measured by different instruments, can be employed as a further approach to assessing the capabilities of each method to accurately measure defects. To use volume and differences between volume measurements as a comparative metric, the volume of a defect is calculated using each point cloud as described above, i.e., that the plane that is defined as the surface is calculated separately for each method using measurements made using that method. These volumes can then be compared to an assigned reference; in this case, the Nikon point cloud and mean differences and statistical measures of these differences can be computed.

Finally, the maximum depth of a defect—and differences in these—is employed as a metric for point cloud comparisons. Using the planes defined by measured points near but outside the defect, the maximum vertical difference between this plane and observed defect points is found. Again, distances can be compared to one another and to an assigned reference. This maximum depth of a defect and statistics about differences tell us about the precisions of the modeling methods and the possible presence of outliers.

#### 2.4.4. Qualitative Experiences in Usability and Efficiency

In addition to computationally comparing the accuracy and precision of modeling methods, it is important to consider other factors such as usability, efficiency, and the user experience. Such considerations are evidently less straightforward and require balancing the values of qualities and properties that are not directly comparable. At the same time, quantitative comparisons of efficiency can also be made, concerning, for example, measurement and processing times. In this study, we provide some referential insight into measurement and processing times, which were not measured or tested robustly, but, more significantly, evaluate the practical implications of different approaches.

## 3. Results

This section presents results of reference measurements, photogrammetric reconstruction, other data processing, and analysis of field measurements.

### 3.1. Reference Measurement Results

**Figure 4:**
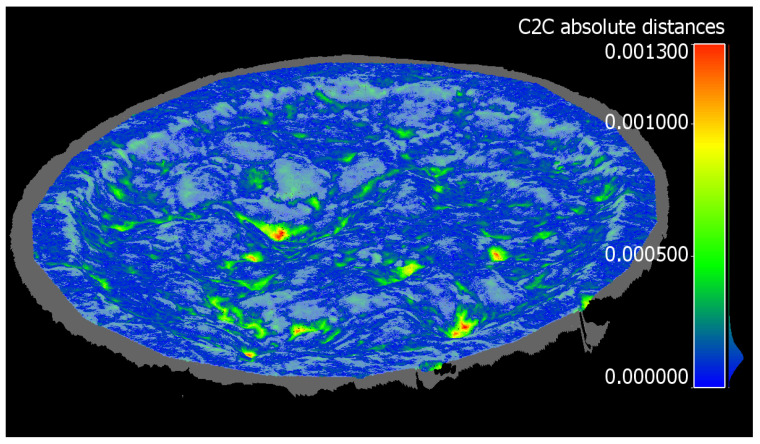
Difference image showing distance to reference model of pavement sample 3, as measured using high-resolution photogrammetry. Reference model is pictured as transparent white.

Mean point–point distances and standard deviations across individual reference samples are presented in Table 8. The table also presents the total number of points, collected by each device, that covers the reference samples. This can be used as a measure of point cloud density. The results are very similar across photogrammetric and Artec Leo point clouds, while TLS is slightly more noisy and the Faro Freestyle stands out as the most noisy. At the same time, the differences in point density stand out relative to positional errors. Notably, the reference measurement setup may favor structured-light scanners, since the samples add geometry to the otherwise flat scene, making tracking more robust. In later analyses, high-resolution photogrammetric point clouds are used as reference due to their robust performance and high point density. While the industrial camera approach obtains even higher density and precision in this comparison, later results show that it can contain inconsistencies (see Section 3.5). Figure 4 shows point–point distance results for Nikon measurements for sample 3, visualizing how the greatest errors are present in the deepest crevices. At most surfaces, errors are practically nonexistent. Relative to the laboratory scan, the Nikon point cloud shows the crevices as more shallow.

### 3.2. Photogrammetric Reconstruction

Table 9 presents the numbers of tie points and reprojection error in photogrammetric processing for high-resolution and industrial camera photogrammetry. The reprojection error metric is dependent on the software used.

### 3.3. Field Measurement Point Clouds

Table 10 and Table 11 provide information on point cloud sizes and densities as produced by different methods. As can be seen, point clouds produced photogrammetrically are much denser than other point clouds. It should be noted that Faro Freestyle point clouds are comparable mainly in terms of point density, since Freestyle measurements only included defective areas of the plots. Grasshopper measurements resulted in the largest point clouds, which is attributable to the large number of images. Point numbers varied especially with handheld scanners, which can at least partially be explained by the measurement mechanism, where a longer view of an area results in more measurements and a denser point cloud. With the Artec Leo, it is also likely that the processing in Artec Studio software influences point density, but the nature of this influence is outside the scope of this study.

For detailed comparisons around defects, a total of 34 defects were identified, sectioned, and examined, and these are detailed in Table 12.

### 3.4. Cross-Section and Graphical Analysis

**Figure 5:**
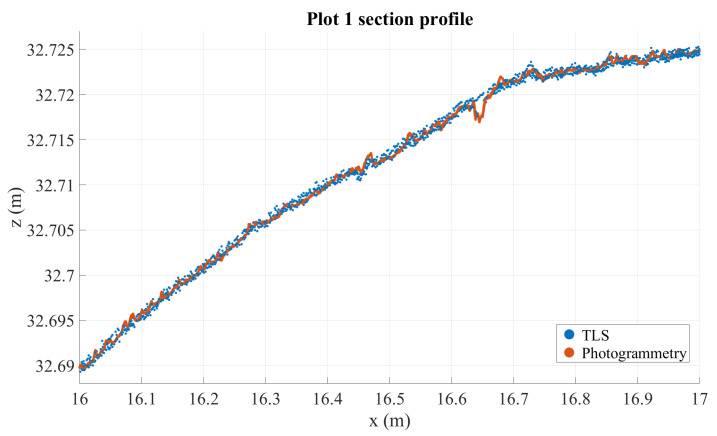
A section of the road profile from plot 1 showing Nikon and TLS point clouds. Photogrammetric data is 1 mm deep, while TLS data is 3 mm deep for visual clarity.

**Figure 6:**
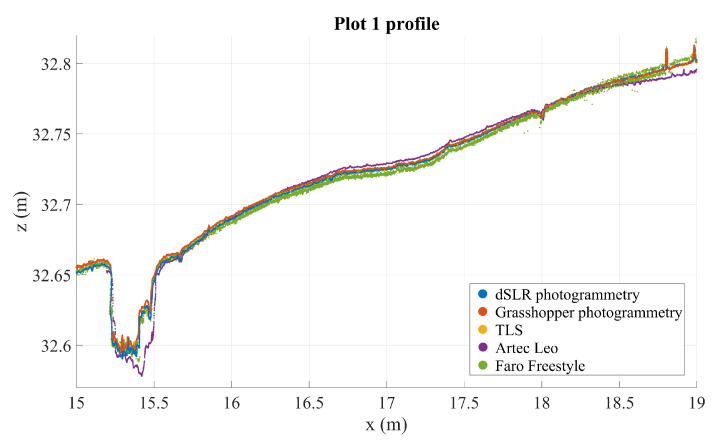
Three mm deep profiles of defect 1 as measured by all instruments in an arbitrary coordinate system.

**Figure 7:**
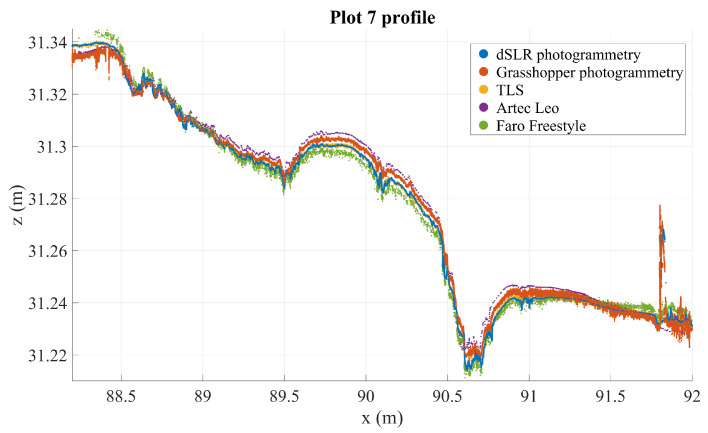
Three mm deep profiles of plot 7 as measured by all instruments in an arbitrary coordinate system.

Cross-section analysis reveals that TLS and Nikon point clouds are the most stable (these are visualized in Figure 5). TLS can be considered precise to a noise level of some millimeters, and similarly accurate. This means that there should be no significant distortion or warping in the road surface as modeled by the TLS point cloud. The profiles in Figure 6 and Figure 7, chosen from two plots with large defects, illustrate how the point clouds retrieved from handheld scanners have tendencies to drift and form hill-like or bowl-like shapes, seen in the middle of the profile where the different point clouds form layers around TLS and Nikon point clouds, according to the amount and direction of distortion. A similar effect is observed with the GH point cloud in plot 7, though not in plot 1 (this likewise occurs in other plots on occasion, but not systematically). In addition, this method appears particularly noisy in plot 7. These observations speak to the inconsistency of the imaging method using the GH (see Section 2.2.2). In both profiles, the Nikon and TLS point clouds align very closely, with TLS appearing slightly more noisy. It is important to remember that the various densities of the point clouds, photogrammetric point cloud densities, are approximately hundredfold relative to other point clouds.

**Figure 8:**
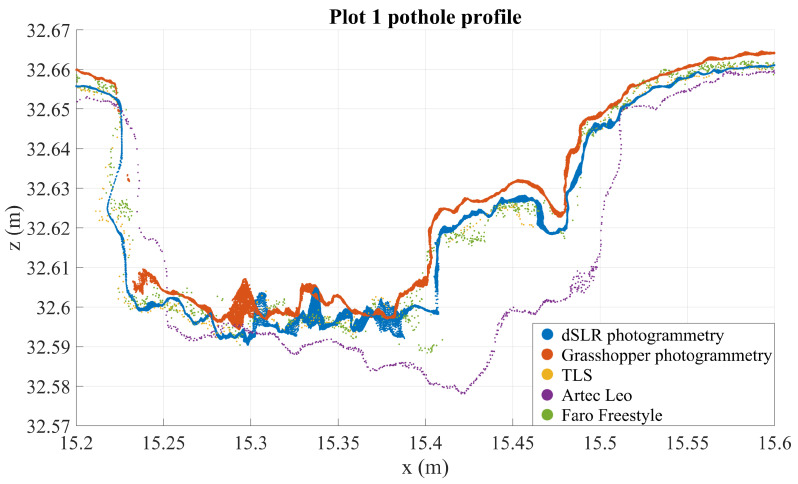
Three mm deep profiles of plot 1 as measured by all instruments in an arbitrary coordinate system.

**Figure 9:**
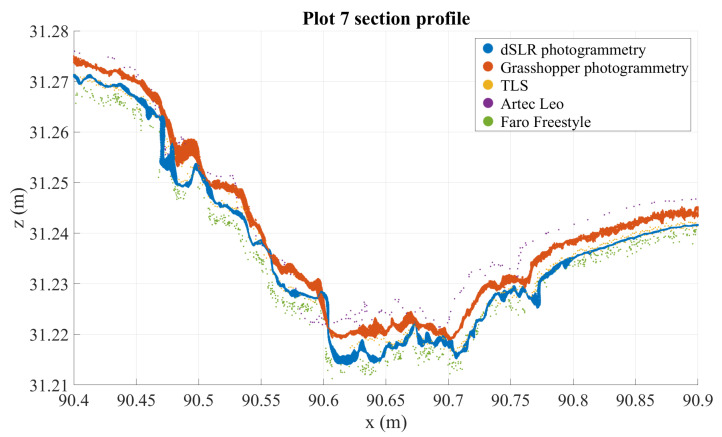
Three mm deep profiles of defect 27 as measured by all instruments in an arbitrary coordinate system.

Figure 5 shows that the photogrammetric point cloud contains little noise as it densely follows a surface. The photogrammetric point cloud reveals much smaller details than does the TLS cloud. In addition, we can see some features in the photogrammetric point cloud that are not clearly visible in the TLS point cloud, for example the crack at 16.65 m on the *x*-axis, which only appears in a few points in the TLS cloud, or the multiple millimeter-sized bumps along the profile. At the same time, many of the shapes in the photogrammetric point cloud can be seen also in the TLS point cloud, if only as individual points slightly offset from the main body of points. For example, the bends at around 16.45–16.48 m—clearly visible in photogrammetric data—appear as a noisy wave in the TLS point cloud as well. The close match of these point clouds sets them apart from the other instruments that contain more noise (the GH and FF point clouds) or distortions (the GH, FF, and Leo point clouds), as can be seen in Figure 6, Figure 7, Figure 8 and Figure 9.

A closer look at individual defects can be seen in Figure 8 and Figure 9, the first of which is a pothole in plot 1 and the second a partially filled crack intersection or pothole in plot 7. The images underline the distortions occurring in some of the point clouds, described above, while also supporting the claim that Nikon point clouds closely follow TLS point clouds while providing higher point density and precision. These images suggest that, of the investigated instruments, photogrammetric and Artec Leo point clouds have sub-millimeter noise levels while TLS and FF point clouds contain noise of some millimeters. At the same time, Figure 8 shows large imprecisions in Leo data, possibly resulting from the temporal difference in measurements, imperfect registration, or warping. TLS and Nikon point clouds contain very little distortion (on this, the TLS point cloud acts referentially), while other methods experience warping at least on occasion. These considerations further support the use of the Nikon point clouds as reference in further point cloud analysis.

### 3.5. Point–Point Distances

For 34 defects, average point–point distances and standard deviations of these between all relevant methods (some defects were outside the measured area of some sensors, in particular the Faro Freestyle) were calculated. Table 13 presents the averages of these averages and standard deviations. As can be seen, values are lowest with high-resolution photogrammetry and TLS.

**Figure 10:**
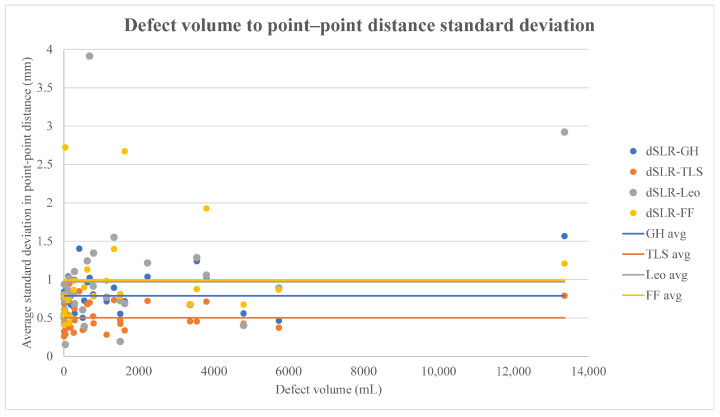
Correlation between defect size and standard deviation in point–point distances.

Further examinations can be made by considering the effect of different kinds of defects on these values. Figure 10 plots point–point distances to defect volume, which illustrates two things and suggests a third: (1) that most defects are small (<1000 mL) in volume; (2) that standard deviations from Nikon data are smallest in TLS, GH, Leo, and FF data, respectively, though the two handheld scanners are quite equal in this regard; (3) that differences between modeling methods seem to be smaller when comparing more voluminous defects. This suggests that stronger geometry results in better models, since larger defects are less planar. This seems plausible especially for the structured-light scanners. However, the amount of data for large defects is notably small and some of the largest deviations happen in the largest defect, so definitive conclusions should not be drawn.

**Figure 11:**
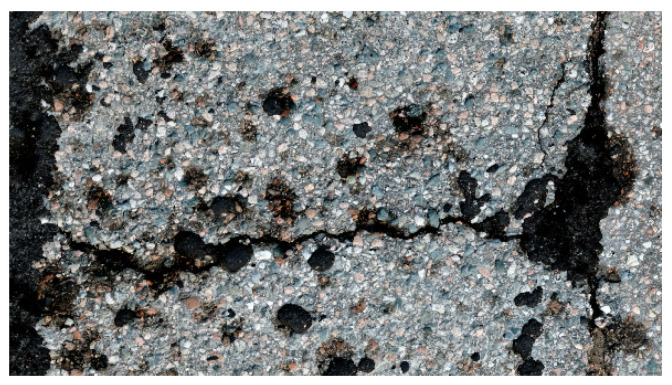
Orthoimage of defect 27 (plot 7).

**Figure 12:**
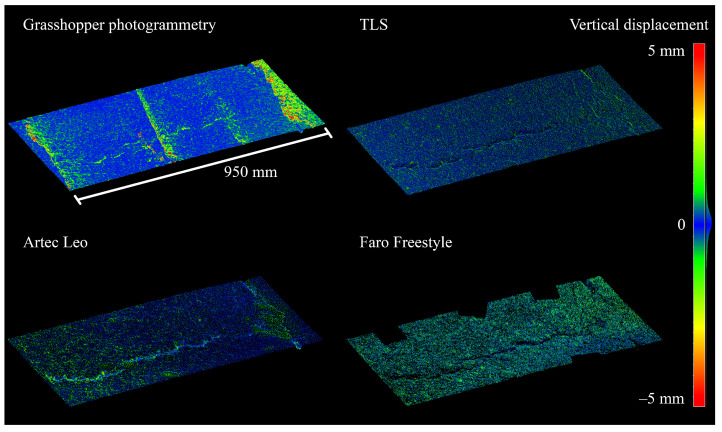
Point clouds of defect 27 (plot 7), colored based on vertical distance to nearest point in Nikon point cloud.

Distances between point clouds can also be interpreted by visualizing pairs of point clouds and differences between these. An example can be found in defect 27, which is pictured in Figure 11 and point clouds of which are visualised in Figure 12, colored based on their vertical distance to the Nikon point cloud. In the figure, it can be seen that there are some noisy areas in the GH point cloud, seemingly a result of the overlapping seams of the back-and-forth imaging process. In the TLS point cloud, there is a small amount of evenly distributed noise, perhaps slightly concentrated in defect areas. The Artec Leo cloud is similar, but the amount of noise is greater and it is less evenly spread. In the Faro Freestyle point cloud, noise seems to be greater, but evenly distributed. This defect was specifically chosen for this image, since it was found to represent the differences between the methods rather well, but similar comparisons were made across different defects.

### 3.6. Volume and Defect Depth

Comparisons of defect volume provide similar results to other comparisons. That is, we find that Nikon and TLS point clouds align best, with GH photogrammetry and handheld scanners following suit. The statistics of volume comparisons (with Nikon measurements as the reference) are in Table 14. The table contains statistics on absolute and relative differences between the methods as compared to Nikon point clouds. Here, relative differences refer to the size of the offset relative to the size of the defect (difference in volume to volume as measured by Nikon cameras). Large standard deviations reveal that there are large differences in volume offsets between defects. That is, none of the methods records the volumes of defects reliably and precisely similarly to high-resolution photogrammetry.

In addition to volume offset, we compare maximum depth as measured by each method. Table 15 presents these results. This comparison reveals the presence of occasional noisy areas in GH point clouds, which have performed better than the handheld scanners so far. This is a result of the same effect that was observed in Figure 12, where “seams” resulting from low overlap in back-and-forth imaging result in noisy areas. It also is evident that the Faro Freestyle point clouds are very noisy, with maximum depths varying significantly from Nikon point clouds.

## 4. Discussion

In order to extract necessary geometric information about various types of pavement distress, the necessary level of detail is highly varied depending on the defect. In this study, some defects were only some milliliters in size (Table 12), while others were multiple liters. While larger defects can be identified in rather low-resolution data of sufficient accuracy, identification and extraction of defect properties, such as depth, width, and volume, requires reliable data of sufficient resolution. To identify and extract information about a 1 mm wide crack, sub-millimeter accuracy is necessary. Potholes, which are typically over 10 cm in diameter and multiple cm deep, can be robustly identified with centimeter-level accuracy. El Issaoui et al. [33] deem a 1.4 mm error level adequate for operational rut depth measurements. For reference use, sub-millimeter levels of accuracy and precision are necessary to reliably evaluate various defect types. As seen in Table 11, of the methods investigated here, only photogrammetric approaches provide this level of detail.

Carefully photographed high-resolution images that are carefully processed into high-density point clouds provide 3D models of higher precision and density than is available from terrestrial or handheld structured-light laser scanning. A lower-resolution industrial camera is also capable of providing high-density models, but imprecisions are more likely to remain, especially when the imaging process is not carefully planned and executed. It is reasonable to assume that a more careful imaging process, perhaps carried out on a moving platform from constant height and with higher overlap would provide results with less noise. TLS modeling is reliable and straightforward, and processing is quick. However, it is insufficient for millimeter-level precision and detail as point densities are very low relative to photogrammetry. While all investigated instrumentations achieved high precision in reference measurements (Section 3.1), photogrammetry delivered unrivaled point density, which is vital for reference use. At the same time, the use of photogrammetry is always a compromise, as choices have to be made about the level of detail and processing settings. It would, for example, be possible to image a research plot with smaller ground sampling distances and larger numbers of images, which might result in higher point densities. In this study, the chosen measurement approaches can be justified as being reasonable for the acquisition of reference data. Closer-range imaging or more delicate measurement conditions would increase the workload in ways that do not reflect realistic measurement conditions.

While photogrammetry seems to perform well, results from handheld structured-light scanners are less straightforward. The measurements used in this study were nonoptimal, with Artec Leo measurements being made 2 months later than other measurements, and Faro Freestyle measurements not covering all defects that were used for comparisons. Some properties of these methods are evident nonetheless. Figure 6, Figure 7 and Figure 12 and Table 15 show that the Freestyle produces very noisy point clouds, which largely disqualifies the scanner from being used to precisely map road defects for reference purposes. The Freestyle also performed poorest in reference measurements. The Leo seems quite capable of producing precise point clouds, but scanning without targets or other ground control points causes drift and bending, as seen in Figure 6 and Figure 7. It was also rather poor at capturing the volume of defects, perhaps because the drift makes creating reference surfaces inaccurate, while obtaining the maximum depth of defects very accurately. Other possible reasons for this are smoothing or hole-filling happening during data processing as a result of some areas being occluded or poorly scanned for other reasons. Another drawback to handheld scanners is that they have limited possibilities for further development, and cannot, for example, be implemented on a mobile platform. With both handheld scanners, point density varied quite a lot, which suggests that human factors in the measurement process—and, with the Artec Leo, the processing process—have a significant effect on this quality. Furthermore, it is unclear whether higher point density—resulting, perhaps, from slower measurements—provides higher accuracy or precision, or whether, indeed, the opposite occurs. The latter could be the case if the tracking of the scanner is noisy. Further investigation into the effects of different measurement techniques with structured-light scanners is required to assess these questions.

### 4.1. Reference Measurements and Systematic Error

Significantly, the largest errors in reference measurements seem to occur at points in the targets where there are the deepest cracks, holes, or crevices (Section 3.1 and Figure 4). Similar results appeared across all instruments. This implies that all of the modeling methods may underestimate defect depth and volume. While the displacement shown in the figure is small, it may have consequences for modeling large, long, or deep cracks, which may as a result be underestimated in size and, therefore, significance, and need repair, especially by algorithmic evaluation. In other words, this suggests that defects are larger than they appear.

### 4.2. Evaluating Usability and Efficiency

#### 4.2.1. Measurement and Processing Times

Table 16 presents approximations of measurement and processing times for a single measurement instance, i.e., plot. It also reports the need for targets in measurement, which contributes to measurement times and preparation needs. These are indicative of what will result in reproductions, but, as will be discussed in Section 4.2, there are many factors affecting these times. Data processing was decentralized across multiple computers with different specifications, making direct comparisons difficult.

The fastest measurements were performed with the handheld Artec Leo scanner, which was able to scan a plot in approximately one or two minutes with practically no preparation. Supplementary scanning (the Artec Leo user interface allows the user to stop and resume scanning) was found to be unhelpful, likely because the simple geometry of the road surface makes it difficult to register multiple scans, compared to one continuous scan. The use of markers, which might improve scanning results with the Leo, would increase preparation time significantly. Nikon imaging took some 5–10 min for a single plot (200–250 images with two cameras). Grasshopper imaging took 1–4 min for actual imaging (500–1600 images per plot at 7 frames per second), but took some additional minutes for setup with a computer and imaging software, focusing, etc. TLS measurements require some 5 min per measurement, and in this study we measured each plot twice, giving a total of 10 min, to which setup times for target spheres, including tripod leveling and GNSS measurements, should be added. Specifying more exact scan areas would reduce this time; that is, not doing complete 360° scans, but only scanning the relevant section. The scanning time on the Faro Freestyle handheld scanner was 1–2 min, making it approximately as fast as the Leo. However, in this study, calibration signals were used to improve the geometry, which increased the total measurement time to 3–4 min, including the placement of the calibration signals, the scanning time, and the removal of the signals. Although only the damaged pavement and surrounding area were scanned with the Freestyle scanner, it would not have taken significantly longer if the entire test area had been scanned.

While the Artec Leo scanned the plot quickly, it required long processing times. The proprietary Artec Studio software that must be used functions similar to a black box, and it is difficult to know what processing is taking place and how much time it requires. Generally, when using nonoptimal settings, a plot was processed in about 5 h. However, using the best possible settings caused extensive processing times (days or weeks), especially in texturing. In this case, where interests are strictly in geometry, texturing could be foregone to accelerate processing, but due to lack of georeferencing in Artec Leo data, texturing (which, in this case, translates to a colored point cloud) made manual registration with other point clouds easier and more reliable. At the same time, improvements in point clouds were hard to find, based on a few computational and visual comparisons. In stark contrast, processing times for individual test plot data from the Faro Freestyle were less than one minute each.

TLS processing required approximately two hours to process all eight plots. Photogrammetric processing times depend on software and hardware being used, number of images, image resolutions, and choices of algorithms for interest point detection, among other factors. A reasonable estimate is that processing a set of images from one research plot into a high-density point cloud takes approximately a day. There are, however, many ways to improve processing times for photogrammetry, such as limiting the number of images, using optimized imaging patterns, using optimal interest point detection, and other algorithms based on the target and limiting the examined area. All measurement methods require a length of time for processing, and in all examined cases, this means hours of passive work. The practical result is that most processing is run overnight, and differences become less consequential. In addition, the time cost is of less importance in the case of reference modeling compared to deployed methods.

#### 4.2.2. Other Considerations

Besides examining the time cost, other factors influence usability and efficiency as well. All discussed methods require a level of sophistication in instrumentation, though the cameras are the most affordable. Collecting images on a dSLR camera is straightforward, though photogrammetric applications require some well-known considerations in imaging, [51,52] (for example). Imaging using the Grasshopper industrial camera as described in Section 2.2.2 is a nonstandard and somewhat complicated approach, and the study of this camera should be considered as a trial for a more systematic approach or rig where several such cameras are installed. As presented, the GH imaging method was unstable and prone to nonoptimal imaging, though it performed well on average. It also results in a deluge of images, most of which do not provide additional information. TLS measurement is straightforward and a well-established surveying method. It requires the use of targets for cloud alignment, but this cost is significant only insofar as it requires more time. The Artec Leo is easy to use and fast to operate, but quality control during operation is challenging, as quite little information is available to the user. Though the instrument notifies the user if tracking is lost, some errors in registration may be revealed only in postprocessing, which is impractical to do onsite. Using targets or signals would make measurement more cumbersome. In processing, the proprietary Artec Studio software is nonideal for research purposes, as the workings of different procedures and algorithms are not clear. Faro Freestyle measurements are quick to produce and process, but seem to be too imprecise and noisy for reliable use in reference modeling. In other use cases, its speed may be a decidingly favorable property as compared to the other instruments discussed here, especially if steps are taken to denoise resulting point clouds. The use of markers is not strictly necessary, but experientially helps prevent drift and distortions.

### 4.3. Future Research

As measurement and modeling technologies continue to develop and improve, further research can provide insight into how new instruments and methodologies compare to existing and established ones. The objective of this study was to determine which method is sufficiently accurate and efficient for use as reference in the study of more versatile pavement defect detection and analysis methods. As such, future research will focus on these methods, which are likely to be mobile and autonomous to varying degrees. Reference measurements remain necessary for benchmarking and evaluation. As research and development are focused mainly on mobile mapping of road defects, reference modeling can be mobilized as well, for example by constructing rigs with high-resolution cameras capable of imaging the entire width of a lane at high resolution. Such rigs can facilitate processing, since initial camera positions can be pre-estimated quite accurately. In addition, they can provide constant quality across different plots as images are not being taken by hand, and alleviate the amount of tedious manual measurement labor in general.

## 5. Conclusions

As the development of increasingly sophisticated defect detection and analysis techniques continues, evaluating these techniques is a necessary component of their development. This research contributes a case study of various state-of-the-art methods for producing close-range, static, high-resolution, three-dimensional reference measurements of pavement defects, investigating three technologies in five instrumentations in real measurement conditions. Such reference measurements can be used to provide ground truth to automated defect detection methods and less-accurate, precise, or dense sensors. The study finds that carefully measured, high-resolution photogrammetric point clouds are the most reliable, detailed, and precise without losing accuracy, providing mean distances between points of down to 0.04 mm and mean accuracies and precisions of 0.2 mm. Industrial camera photogrammetry can provide similar densities, accuracy and precision, but due to the imaging method deployed here, can retain distinct erroneous areas. Terrestrial laser scanning is likewise accurate, but much less dense. Of the investigated handheld structured-light scanners, the Artec Leo provides high accuracy and precision but only on a small scale, and has much lower point density (1–10 mm between points on average), while the Faro Freestyle creates quite noisy point clouds (almost 1-millimeter errors in reference measurements) with only slightly better densities than the Leo. While the photogrammetric approaches were superior in density, accuracy, and precision, other factors, such as measurement and processing time cost, may favor the other approaches. TLS holds the middle ground for both accuracy and precision and efficiency concerns, while the handheld scanners provide quick measurements and, specifically in the case of the Faro Freestyle, processing. Due to the requirements for reference measurements in developing and evaluating defect detection methods, photogrammetric or TLS approaches provide the most reliable reference datasets, with the choice depending mainly on required level of detail.

## Figures and Tables

**Table 1:** Pavement distress types [5].

Distress Type	Mechanism	Manifestation
Cracking	Fractures of surface and fundamental pavement layers	Transverse, longitudinal, edge, block, alligator cracks
Disintegration	Progressive division of pavement into loose pieces	Potholes, patches
Surface deformation	Change in pavement structure	Shoving, rutting, distortion
Surface defects	Loss of surface microtexture or macrotexture	Raveling, bleeding

**Table 2:** Test plot locations.

Plot Number	Location (ETRS-TM35FIN)
1	(363545, 6672374)
2	(363602, 6672639)
3	(363634, 6672740)
4	(363659, 6672781)
5	(363723, 6672860)
6	(363801, 6672933)
7	(363995, 6673252)
8	(363955, 6673569)

**Table 3:** Camera settings.

	Nikon D800E	Nikon D810
	24 mm Lens	50/60 mm Lens
Aperture	8	14
ISO	100–800	800–1600
Shutter speed	1/80–1/125 s	1/125 s

**Table 4:** RealityCapture image alignment and processing settings.

Alignment Settings	
Engine	RealityCapture
Mode	High
Max features per Mpx	0
Max features per image	0
Detector sensitivity	Medium
Preselector features	10,000
Image downscale factor	1
Maximal feature reprojection error [pixels]	3.00
Use camera positions	True
Lens distortion model	K + Brown3 with tangential2
Final optimization	True
**Model Generation Settings**	
Quality level	High

**Table 5:** Agisoft Metashape image alignment and processing settings.

Alignment Settings	
Accuracy	Highest
Generic preselection	Yes
Reference preselection	Sequential
Key point limit	0
Tie point limit	0
Filter points by mask	No
Mask tie points	No
Guided image matching	No
Adaptive camera model fitting	Yes
**Depth Map Processing Settings**	
Quality	Ultra High
Filtering mode	Aggressive

**Table 6:** Artec Leo and Faro Freestyle specifications as provided by the manufacturers. All information is not provided for both. RMS: root mean square; VCSEL: vertical cavity surface-emitting laser; LED: light-emitting diode; DOF: degree of freedom.

Specification	Artec Leo	Faro Freestyle
3D point accuracy	0.1 mm	0.5 mm
3D point resolution	0.2 mm	0.2 mm
RMS noise @ 0.8 m range	–	0.8 mm
Working distance	0.35–1.2 m	0.5–3 m
3D reconstruction rate	22 fps	–
Data acquisition speed	35 Mpts/s	88 kpts/s
3D light source	VCSEL	LED flash
Position sensing	9-DOF inertial system	–

**Table 7:** Pavement samples used for reference measurements.

ID	Diameter (mm)	Height (mm)	Description
1	102	31	Fresh, bituminous and coarse pavement
2	102	29	Heavily worn, low bitumen
3	102	29	Heavily worn and cracked
4	95	62	Slightly worn, large grain
5	153	59	Artificially smooth

**Table 8:** Reference measurement results and point cloud sizes, where *s* is the mean distance to nearest point for reference blocks separately and together, σ is standard deviation, and *n* the number of points. Nikon: high-resolution photogrammetry; GH: industrial camera photogrammetry; TLS: terrestrial laser scanning; Leo: Artec Leo; FF: Faro Freestyle.

	Nikon	GH	TLS	Leo	FF
*n*	934,097	1,087,748	50,585	4480	8183
$s_{1}$ (mm)	0.34	0.25	0.43	0.30	1.22
$s_{2}$ (mm)	0.39	0.15	0.41	0.18	0.98
$s_{3}$ (mm)	0.15	0.18	0.41	0.20	0.65
$s_{4}$ (mm)	0.13	0.15	0.35	0.15	0.49
$s_{5}$ (mm)	0.20	0.20	0.37	0.20	0.49
$\sigma_{1}$ (mm)	0.24	0.19	0.31	0.21	0.74
$\sigma_{2}$ (mm)	0.26	0.11	0.35	0.13	0.54
$\sigma_{3}$ (mm)	0.10	0.14	0.31	0.14	0.46
$\sigma_{4}$ (mm)	0.06	0.08	0.29	0.08	0.38
$\sigma_{5}$ (mm)	0.08	0.07	0.22	0.10	0.33
$s_{mean}$ (mm)	0.24	0.19	0.40	0.20	0.77
$\sigma_{mean}$ (mm)	0.15	0.12	0.30	0.13	0.48

**Table 9:** Photogrammetric reconstruction results for Nikon and GH measurements for each plot.

Plot Number	Tie Points (Nikon)	Nikon Mean Reprojection Error (Pixels)	Tie Points (GH)	GH RMS Reprojection Error (Pixels)
1	9,708,844	0.39	10,144,098	0.33
2	13,903,757	0.38	3,203,031	0.54
3	15,743,599	0.46	15,101,018	0.35
4	11,888,215	0.43	15,381,474	0.47
5	9,893,413	0.51	12,773,785	0.54
6	16,196,109	0.43	4,469,455	0.71
7	14,784,526	0.39	11,174,053	0.47
8	15,778,640	0.43	15,778,194	0.42

**Table 10:** Final point cloud sizes as produced by different instrumentations, cropped to the research plot. Due to misalignment, GH measurements for plot 5 were discarded.

Plot Number	Nikon	GH	TLS	Leo	Freestyle
1	140,396,091	310,755,752	6,389,651	6,001,849	11,463,027
2	287,491,907	335,736,791	3,534,097	18,146,934	3,447,980
3	252,277,528	327,961,220	5,101,989	6,009,833	2,522,223
4	255,212,221	650,398,114	6,126,542	1,626,388	3,718,867
5	260,599,273	–	7,157,959	2,013,197	2,984,767
6	238,366,658	535,690,203	7,336,520	1,616,939	7,336,520
7	216,517,583	487,028,634	7,252,653	2,009,979	2,045,904
8	238,496,328	455,029,861	8,396,739	2,005,995	791,550

**Table 11:** Point cloud density expressed as mean horizontal distance to nearest point in millimetres.

Plot Number	Nikon	GH	TLS	Leo	Freestyle
1	0.11	0.04	2.01	3.22	0.92
2	0.05	0.05	6.46	0.85	1.43
3	0.05	0.06	7.43	3.17	1.37
4	0.05	0.03	3.81	7.80	1.54
5	0.06	–	2.59	6.64	1.38
6	0.05	0.03	2.17	9.03	1.40
7	0.04	0.02	2.61	7.40	1.94
8	0.05	0.02	3.11	9.69	6.54

**Table 12:** Segmented individual defects and some of their properties. Defects 8 and 30 were discarded during processing. Volume computation is described in Section 2.4.3.

Defect Number	Plot Number	Description	Volume (mL)
1	1	pothole	13,361
2	1	longitudinal crack	1141
3	1	longitudinal crack & small pothole	3547
4	2	longitudinal crack	3806
5	2	longitudinal crack	626
6	2	alligator crack	1342
7	2	transverse crack	288
9	4	longitudinal crack	11
10	4	longitudinal crack	119
11	4	longitudinal crack	264
12	4	longitudinal crack	286
13	4	longitudinal crack	414
14	5	transverse crack	16
15	5	transverse crack	10
16	5	crack	151
17	5	raveling	3371
18	6	longitudinal crack	792
19	6	crack	19
20	6	crack	23
21	6	longitudinal crack	10
22	6	long central line pothole	2233
23	6	longitudinal crack	687
24	7	transverse crack	7
25	7	pothole and transverse crack	1504
26	7	longitudinal crack	141
27	7	longitudinal crack & filled pothole	543
28	7	wide longitudinal crack	5739
29	7	longitudinal crack	180
31	8	longitudinal/alligator crack	1627
32	8	longitudinal crack	40
33	8	longitudinal crack	507
34	3	crack network	4796
35	3	raveling	1506
36	3	wide longitudinal crack	784

**Table 13:** Average mean point–point distances and standard deviations as compared across all methods. Numbers are in millimetres.

		Nikon	GH	TLS	Leo	FF
**Nikon**	mean		0.829	0.595	1.026	1.061
	std dev		0.787	0.502	0.969	0.993
**GH**	mean	0.829		0.613	1.005	0.994
	std dev	0.787		0.529	0.972	1.016
**TLS**	mean	0.595	0.613		1.458	1.429
	std dev	0.502	0.529		0.930	0.928
**Leo**	mean	1.026	1.005	1.458		1.840
	std dev	0.969	0.972	0.930		1.109
**FF**	mean	1.061	0.994	1.429	1.840	
	std dev	0.993	1.016	0.928	1.109	
**mean**	mean	0.878	0.860	1.024	1.332	1.331
	std dev	0.813	0.826	0.722	0.995	1.012

**Table 14:** Offsets and standard deviation of offsets of defect volumes compared to volume measured from Nikon point cloud.

Method	Mean Offset (mL)	Standard Deviation (mL)	Mean Relative Offset (%)	Std. Dev of Relative Offset (%)
GH	145	356	5.8	18.7
TLS	31	183	3.7	15.6
Leo	189	530	5.4	32.8
FF	−103	539	−15.4	25.2

**Table 15:** Offsets and standard deviations of offsets of defect maximum depth as compared to maximum depth measured from Nikon point clouds. Relative values are excluded due to large variety in defects’ depth but comparatively constant values in offset, which lead to large relative offsets that do not reflect real inaccuracies.

Method	Mean Offset (mm)	Standard Deviation (mm)
GH	4.0	5.0
TLS	0.7	1.1
Leo	1.9	2.9
FF	7.0	22.4

**Table 16:** Measurement and processing times and target requirements for each method. The numbers are approximate and referential, and real results will differ with circumstances.

Method	Measuring Time (Minutes)	Processing Time (Minutes)	Requires Targets or Markers
Nikon	10	1400	For scale
GH	5	1400	For scale
TLS	20	120	For registration
Leo	2	300	No
FF	4	1	For drift prevention

## Data Availability

The pavement sample measurement data presented in this study are openly available in Zenodo [53].
